# Three New Species of *Aceria* (Acari: Trombidiformes: Eriophyoidea) from China †

**DOI:** 10.3390/ani14050720

**Published:** 2024-02-25

**Authors:** Mengchao Tan, Ranran Lian, Hongyan Ruan, Xuhui Liang

**Affiliations:** 1 Key Laboratory of Beibu Gulf Environment Change and Resources Utilization of Ministry of Education, Nanning Normal University, Nanning 530001, China; rhyan@nnnu.edu.cn; 2 Guangxi Key Laboratory of Agric-Environment and Agric-Products Safety, National Demonstration Center for Experimental Plant Science Education, College of Agriculture, Guangxi University, Nanning 530004, China; 18939601915@163.com (R.L.); 17776712629@163.com (X.L.)

**Keywords:** Aceriini, Eriophyidae, Guangxi, taxonomy, galls

## Abstract

**Simple Summary:**

The superfamily Eriophyoidea includes more than 5000 species worldwide and is a group of phytophagous mites that has an important influence on the agricultural economy. Aceria is a rich genus of more than 1000 species that belongs to the family Eriophyidae, which is distributed throughout the whole world. Here, three new species, *Aceria bischofiae* **sp. nov.**, *Aceria cryptocaryae* **sp. nov.,** and *Aceria buddlejae* **sp. nov.,** from Guangxi and Chongqing Province, China (the Oriental realm), are described and illustrated.

**Abstract:**

Three new *Aceria* species from South China are described and illustrated. *Aceria bischofiae* **sp. nov.** was collected on *Bischofia javanica* Blume (Phyllanthaceae), inducing galls on surfaces of the leaves; *Aceria cryptocaryae* **sp. nov.** was collected on *Cryptocarya metcalfiana* Allen (Lauraceae), causing the formation of erinea on the undersurface of the leaves; and *Aceria buddlejae* **sp. nov.** was collected as a vagrant on *Buddleja lindleyana* Fort. (Scrophulariaceae) leaves, and no symptoms were observed on the host plant.

## 1. Introduction

Eriophyoidea (Acari: Prostigmata) is a large mite superfamily and among the smallest arthropods known. Until now, more than 5000 named species have been recognized, some of which are significant pests of agronomic plants [1,2], and over 80% of eriophyoid mite species are monophagous, registered on only one host plant [3,4]. Host plants supposedly played key roles in their diversification [5].

*Aceria* Keifer is the genus of the family Eriophyidae Nalepa with the highest number of known species. Until now, more than 1000 species names of *Aceria* have been reported around the world, of which about 81 species have been found in China [6,7,8,9,10]. However, some species within *Aceria* are described too simply, and their taxonomic status needs to be further clarified through more detailed morphological descriptions and comprehensive taxonomic methods.

*Bischofia javanica* Blume is an evergreen tree belonging to the family Phyllanthaceae, broadly distributed in China, India, Bangladesh, and Southeast Asia. The nutritional value of this plant is very high, and the leaves are widely used in the preparation of salads and condiments [11]. Until now, three eriophyoid mites have been described from the plants of the genus *Bischofia*: *Phyllocoptruta maerimae* Boczek and Chandrapatya, 2000; *Bischofius kanchanaburi* Boczek and Chandrapatya, 2000; and *Diptilomiopus bischofiae* Li, Wei, and Wang, 2009 [12,13]. *Buddleja lindleyana* Fort. is a garden ornamental plant and also a commonly used medicinal plant that belongs to the family Scrophulariaceae, which is native to China and mainly distributed in most parts of southern China. It is also distributed in America, Malaysia, Africa, and so on [14]. To date, one eriophyoid mite has been described from the plants of the genus *Buddleja*: *Aculops salviifoliae* Meyer and Ueckermann, 1990 [15]. *Cryptocarya metcalfiana* Allen belongs to the family Lauraceae, which is distributed in South China. Only one eriophyoid mite has been described from the plants of the genus *Cryptocarya*: *Aceria aphanothrix* (Nalepa, 1923) [16].

This paper presents descriptions of three new *Aceria* species: *Aceria bischofiae* **sp. nov.**, *Aceria cryptocaryae* **sp. nov.**, and *Aceria buddlejae* **sp. nov.** from the subtropical zone of China (the Oriental Region).

## 2. Materials and Methods

Mite specimens were collected from different host plants in Guangxi and Chongqing provinces by the aid of a hand lens (80×) (brand: Binyun; model: BY2600; manufacturer: Xinxiang Optics, Hangzhou, China) in China. The mites were collected from leaf samples and stored in a 70% ethanol solution using a brush. Samples were slide-mounted in modified Berlese medium [17] without adding additional fibers [18]. All specimens were examined with an Olympus CX41 (Philippines) microscope under phase contrast (oil immersion: 100×/1.25; widefield eyepiece: 10×). Micrographs were obtained from a Nikon DS-Ri2 microscope. The morphological terminology used in the morphological description of the mites follows Lindquist [1] and Amrine et al. [19], and internal female genitalia nomenclature follows Chetverikov [20]. The generic classification follows Amrine et al. [19] in combination with descriptions of all the published genera after 2003. All morphological measurements were according to Amrine and Manson [17], as modified by de Lillo et al. [18]. Measurements refer to the length of the morphological trait unless otherwise specified and are given in micrometers (μm). The holotype female measurement precedes the corresponding range for paratypes (given in parentheses). For males, only the ranges are given. Moreover, “*” in the descriptions means there is no variation in measurements. The number of measured specimens (n) is given within parentheses in the description of each stage. Line drawings were prepared according to de Lillo et al. [18], and abbreviations used in figures follow Amrine et al. [19]. Host plant names and their synonymies are in accordance with The World Flora Online “http://www.worldfloraonline.org/” (30 May 2023).

Type materials are deposited at the Key Laboratory of Beibu Gulf Environment Change and Resources Utilization of the Ministry of Education, Nanning Normal University, Guangxi, China.

## 3. Results

### Systematics

Family: Eriophyidae Nalepa, 1898.

Subfamily: Eriophyinae Nalepa, 1898.

Tribe: Aceriini Amrine and Stasny, 1994.

Genus: *Aceria* Keifer, 1944.

**Figure 1:**
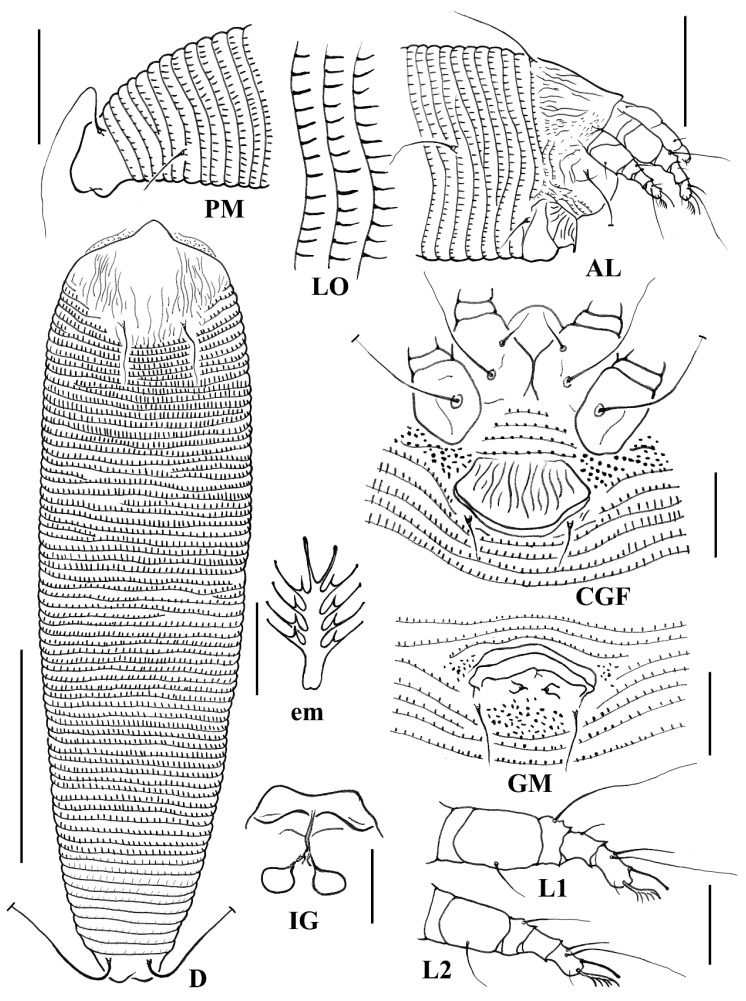
Line drawings of *Aceria bischofiae* **sp. nov.**: **AL**. Lateral view of anterior opisthosoma; **CGF**. Coxigenital region of female; **D**. Dorsal view; **em**. Empodium; **GM**. Male genitalia; **IG**. Internal female genitalia; **LO**. Lateral view of annuli; **L1**. Leg I; **L2**. Leg II; **PM**. Lateral view of the posterior opisthosoma. Scale bar: 50 μm for **D**; 25 μm for **AL** and **PM**; 10 μm for **CGF**, **GM**, **LO**, **L1**, **L2**, and **IG**; 2.5 μm for **em**.

**Figure 2:**
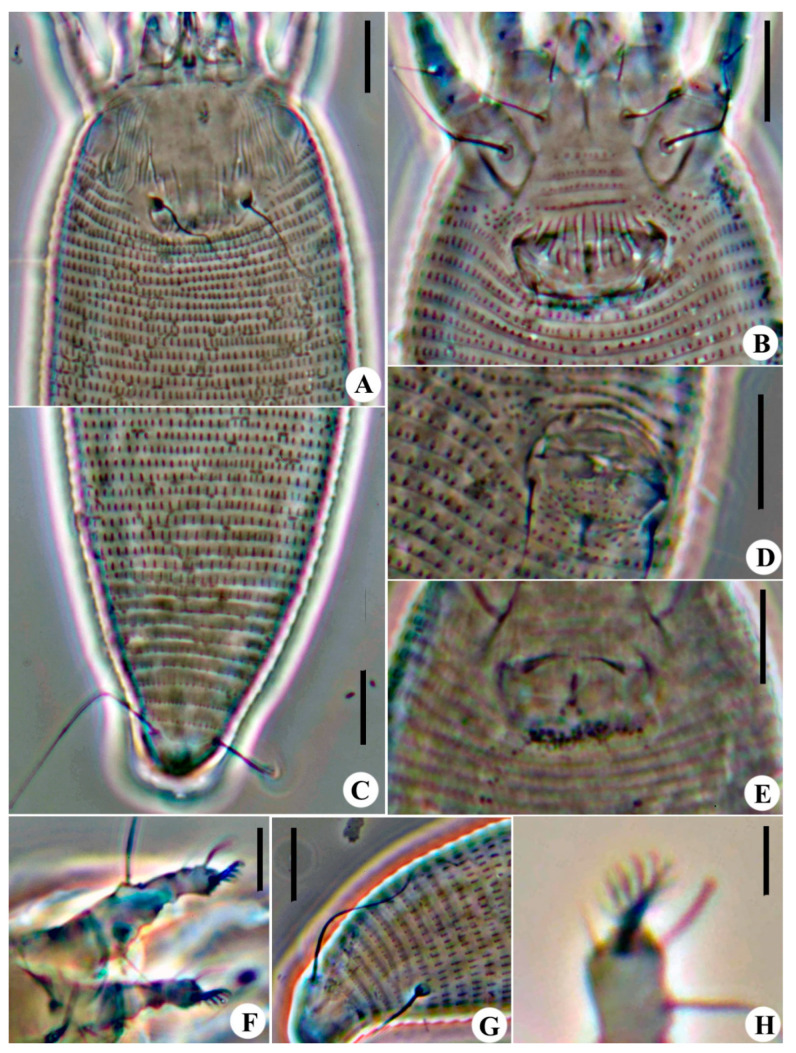
Images of *Aceria bischofiae* **sp. nov.**: (**A**) Prodorsal shield of female; (**B**) Female coxigenital area; (**C**) Postero-dorsal view of mite; (**D**) Male coxigenital area; (**E**) Internal genitalia; (**F**) Legs; (**G**) Postero-lateral view of mite; (**H**) Empodium. Scale bar: 10 μm for (**A**–**G**); 2.5 μm for (**H**).

***Aceria bischofiae* sp. nov. (Figure 1 and Figure 2)**

Description: **Female** (n = 15). Body vermiform, 191 (185–202, including gnathosoma), 48 (43–48) wide, 44 (42–46) thick. **Gnathosoma** 19 (18–21), projecting obliquely downwards, pedipalp coxal setae *ep* 2 (2–3), dorsal pedipalp genual setae *d* 3 (2–4), unbranched, palp tarsus setae *v* 1 (1–2), cheliceral stylets 18 (18–20). **Prodorsal shield** 29 (27–30), including frontal lobe, 38 (33–38) wide; with a short flexible distally rounded frontal lobe, 3 (3–5), over gnathosomal base. Median lines and admedian lines are present on the posterior half of the shield; submedian lines do not reach the rear shield margin; a few short dashes medially; and some short and long dashes on the lateral margin of the shield. Tubercles of scapular setae *sc* on rear shield margin, 14 (12–15) apart, scapular setae *sc* 20 (18–21), divergently backward. **Coxae** smooth; prosternal apodeme 5 (5–6); setae *1b* 7 (7–8), tubercles *1b* 7 (6–7) apart; setae *1a* 16 (15–20), tubercles *1a* 8 (8–9) apart; setae *2a* 29 (27–33), tubercles *2a* 17 (16–18) apart. **Leg I** 24 (20–25), femur 8 (7–8), femoral setae *bv* 7 (5–7), genu 3 (3–4), genual setae *l*″ 18 (16–18), tibia 4 (4–5), tibial setae *l*′ 2 (2–3), tarsus 5 (5–6), tarsal setae *ft*′ 6 (5–8), setae *ft*″ 15 (11–16), setae *u*′ 3 (2–3), solenidion *ω* 5 (5–6), curved down, distally simple, empodium simple, 4 (4–5), 5-rayed. **Leg II** 22 (20–23), femur 7 (7–8), femoral setae *bv* 6 (6–8), genu 3 (3–4), genual setae *l*″ 8 (5–8), tibia 3 (3–4), tarsus 4 (4–5), tarsal setae *ft*′ 5 (4–5), setae *ft*″ 12 (10–12), setae *u*′ 3 (3–4), solenidion *ω* 6 (5–6), curved down, distally simple, empodium simple, 4 (4–5), 5-rayed. **Opisthosoma** with 68 (67–70) dorsal semiannuli, with elongate microtubercles, and 62 (61–64) ventral semiannuli, with small elongate microtubercles on rear annulus margin; coxigenital region with 4 (3–4) semiannuli between coxae and genitalia, with fine microtubercles; last 8 (8–9) dorsal semiannuli with fine and elongated microtubercles. Setae *c2* 20 (19–21), on ventral semiannulus 11 (10–11), 40 (38–44) apart; setae *d* 31 (28–33), on ventral semiannulus 23 (22–23), 31 (30–33) apart; setae *e* 38 (36–39), on ventral semiannulus 38 (38–39), 18 (15–19) apart; setae *f* 13 (11–14), on 6th ventral semiannulus from rear, 12 (11–13) apart. Setae *h2* 38 (33–40), setae *h1* absent. **Genital coverflap** 11 (10–12), 18 (17–18) wide, coverflap with 15 (14–16) longitudinal ridges, setae *3a* 7 (5–7), 13 (12–13) apart. **Internal female genitalia**, spermathecae ovoid, oriented posterolateral; spermathecal tubes relatively short; short spermathecal tubes, directed laterad; transverse genital apodeme trapezoidal, distally folded.

**Male** (n = 3). Similar in shape and prodorsal shield arrangement to female. Body 175–182, 40–41 wide. **Gnathosoma** 18–19, projecting obliquely downwards, setae *ep* 2*, setae *d* 2–3, unbranched, setae *v* 1*, cheliceral stylets 18*. **Prodorsal shield** 25–26, 30* wide. Tubercles of scapular setae *sc* on rear shield margin, 13* apart, scapular setae *sc* 18–19, divergently backward. **Coxae** smooth; setae *1b* 7–8, tubercles *1b* 6* apart; setae *1a* 16–18, tubercles *1a* 8* apart; setae *2a* 30–32, tubercles *2a* 17* apart. **Leg I** 22–24, femur 7–8, femoral setae *bv* 6–7, genu 3*, genual setae *l*″ 16–18, tibia 4*, tibial setae *l*′ 2*, tarsus 4–5, tarsal setae *ft*′ 5–6, setae *ft*″ 14–16, setae *u*′ 3*, solenidion *ω* 5*, curved down, distally simple, empodium simple, 5*, 4-rayed. **Leg II** 20–22, femur 7–8, femoral setae *bv* 6–7, genu 3*, genual setae *l*″ 7–8, tibia 3*, tarsus 4–5, tarsal setae *ft*′ 4*, setae *ft*″ 12 10–11, setae *u*′ 2*, solenidion *ω* 6*, curved down, distally simple, empodium simple, 5*, 4-rayed. **Opisthosoma** dorsally arched with 67–68 dorsal semiannuli, with elongate microtubercles, and 63–65 ventral semiannuli, with elongate microtubercles on the rear annulus margin; coxigenital region with 3* semiannuli between coxae and genitalia, with fine microtubercles. Setae *c2* 17–19, on ventral semiannulus 10*, 40–42 apart; setae *d* 30–34, on ventral semiannulus 22*, 30–31 apart; setae *e* 32–37, on ventral semiannulus 37*, 17* apart; setae *f* 12*, on 6th ventral semiannulus from rear, 11* apart. Setae *h2* 35–38, setae *h1* absent. **Genitalia** 9–11, 13–15 wide, setae *3a* 6–8, 11–12 apart.

**Type material:** Holotype, female (slide number EAA2-3.1; marked Holotype), found on *Bischofia javanica* Blume (Fam. Phyllanthaceae), Nanning Normal University, Nanning City, Guangxi, China, 23°10′55″ N, 108°17′12″ E, elevation 109 m, 23 May 2023, coll. Meng-Chao Tan. Paratypes, 14 females on 14 slides and three males on three slides (slide number EAA2-3.2~3.18; marked Paratypes), from *B. javanica*, with the same details as holotype.

**Type of host plant:** *Bischofia javanica* Blume (Fam. Phyllanthaceae).

**Figure 3:**
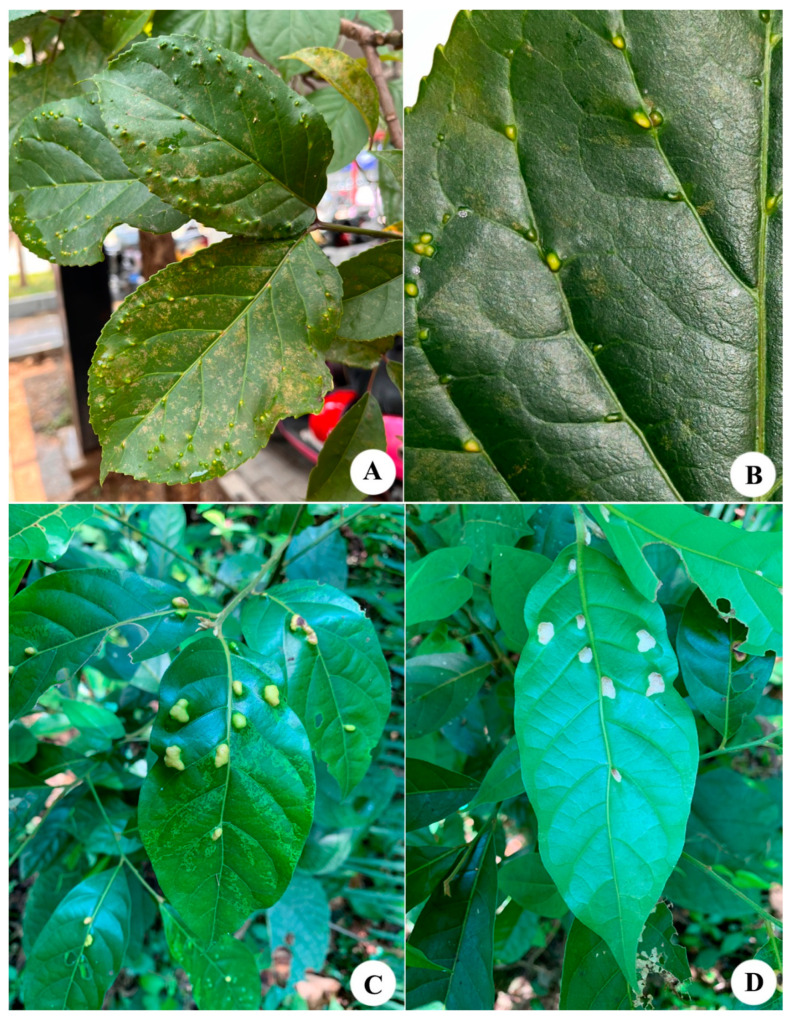
(**A**,**B**) Damage symptoms associated with *Aceria bischofiae* **sp. nov.** on *Bischofia javanica* Blume; (**C**,**D**) Erineum caused by *Aceria cryptocaryae* **sp. nov.** on *Cryptocarya metcalfiana* Allen.

**Relation to the host plant:** mites induce small round galls on the surfaces of the leaves (Figure 3A,B).

**Etymology:** the species is named after the generic name of the type of host plant, i.e., *Bischofia,* in the genitive case.

**Differential diagnosis:** *Aceria bischofiae* **sp. nov.** appears to be close to *Aceria varia* (Nalepa, 1892), which was originally found on *Populus tremula* L. (Salicaceae) in France and Iran [21,22]. *Aceria bischofiae* **sp. nov.** and *A. varia* have similar short median line at the basal third of shield, numerous short lines on the outer side of the shield, empodium 5-rayed, genital coverflap with 14–16 longitudinal ridges, but they differ by the number of rings of the opisthosoma (67–70 dorsal semiannuli and 61–64 ventral semiannuli in *A. bischofiae* **sp. nov.** versus 73–86 dorsal semiannuli and 64–80 ventral semiannuli in *A. varia*), setae *h1* (absent in *A. bischofiae* **sp. nov.** versus 9–10 in *A. varia*), the coxal ornamentation (smooth in *A. bischofiae* **sp. nov.** versus with distinct granules in *A. varia*), the length of the scapular setae *sc* (18–21 μm in ***A.*** *bischofiae* **sp. nov.** versus 31–35 μm in *A. varia*), the length of the scapular setae *d* and setae *e* (setae *d* 28–33 μm, setae *e* 36–39 μm in *A. bischofiae* **sp. nov.** versus with setae *d* 46–60 μm, setae *e* 15–17 μm in *A. varia*).

This new species also has few morphological similarities to *Aceria lagerstroemiae* Kuang and Yang, 1994, collected on *Lagerstroemia indica* L. in China [23], including coxal ornamentation (with 15–18 longitudinal ridges), coxae smooth, setae *h1* absent, number of dorsal semiannuli (65–70), scapular setae *sc* length, as well as the length of ventral setae *d*, *e,* and *f*. The new species can be differentiated for prodorsal shield ornamentation (median line and admedian lines present on about posterior half of the shield, a few short dashes medially and some short and long dashes on the lateral margin of the shield in *A. bischofiae* **sp. nov.** versus shield ornamented several lines in *A. lagerstroemiae*), the number of empodium rays (5-rayed in *A. bischofiae* **sp. nov.** versus 6-rayed in *A. lagerstroemiae*), and the shape of microtubercles on dorsal semiannuli (with elongate microtubercles in *A. bischofiae* **sp. nov.** versus with semi-oval microtubercles in *A. lagerstroemiae*).

**Figure 4:**
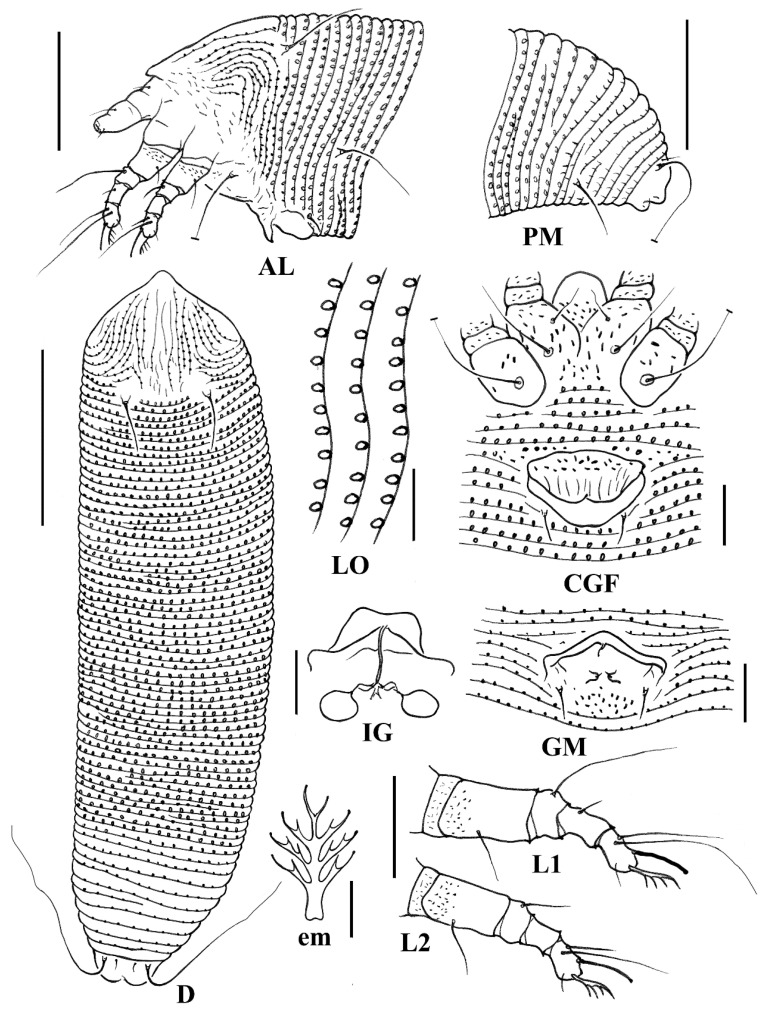
Line drawings of *Aceria cryptocaryae* **sp. nov.**: **AL**. Lateral view of anterior opisthosoma; **CGF**. Coxigenital region of female; **D**. Dorsal view; **em**. Empodium; **GM**. Male genitalia; **IG**. Internal female genitalia; **LO**. Lateral view of annuli; **L1**. Leg I; **L2**. Leg II; **PM**. Lateral view of the posterior opisthosoma. Scale bar: 50 μm for **D**; 25 μm for **AL** and **PM**; 10 μm for **CGF**, **GM**, **LO**, **L1**, **L2**, and **IG**; 2.5 μm for **em**.

**Figure 5:**
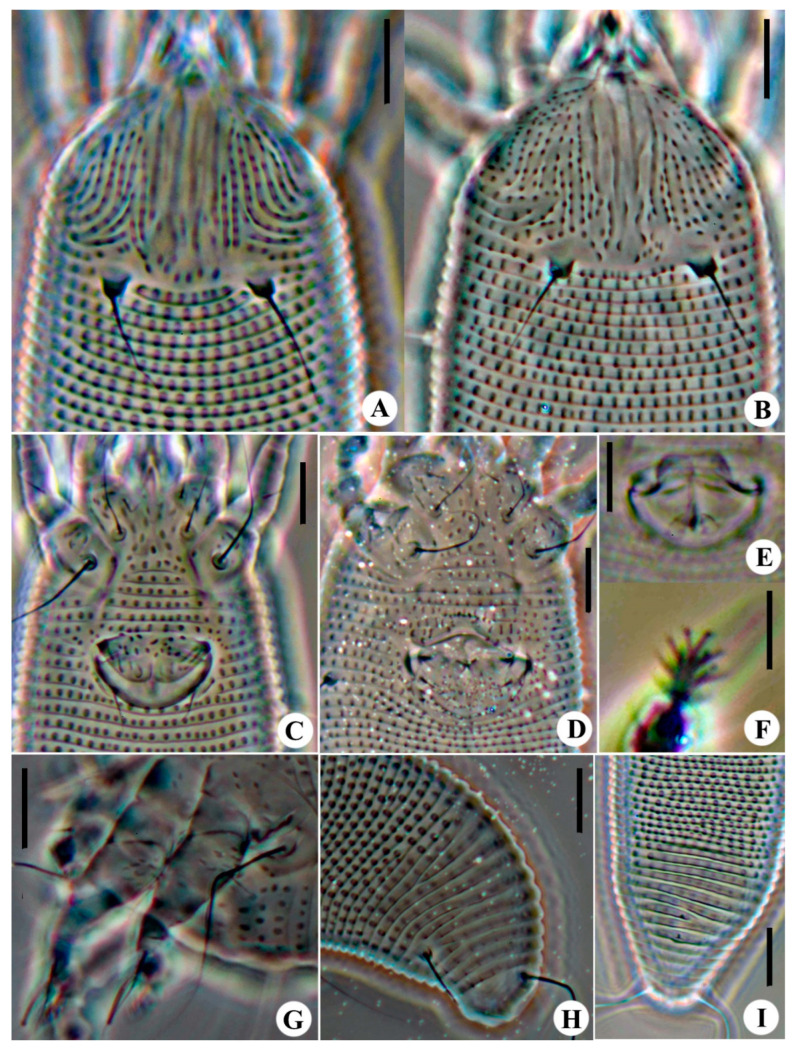
Images of *Aceria cryptocaryae* **sp. nov.**: (**A**) Prodorsal shield of female; (**B**) Prodorsal shield of male; (**C**) Female coxigenital area; (**D**) Male coxigenital area; (**E**) Internal genitalia; (**F**) Empodium; (**G**) Legs; (**H**) Postero-lateral view of mite; (**I**) Postero-dorsal view of mite. Scale bar: 10 μm for (**A**–**E**,**G**–**I**); 5 μm for (**F**).

***Aceria cryptocaryae* sp. nov. (Figure 4 and Figure 5)**

Description: **Female** (n = 15). Body vermiform, 199 (193–231, including gnathosoma), 53 (48–53) wide, 48 (45–48) thick. **Gnathosoma** 18 (18–20), projecting obliquely downwards, setae *ep* 2 (2–3), setae *d* 3 (3–4), unbranched, setae *v* 1 (1–2), cheliceral stylets 16 (15–16). **Prodorsal shield** 26 (25–26), including frontal lobe, 31 (29–33) wide. The shield pattern is distinct and composed of granules aligned and connected by lines as follows: an incomplete median line broken; two complete, sinuous subparallel admedian lines; diverging posteriorly; submedian lines incomplete, extending from the anterior margin and ending ahead of the prodorsal shield tubercle; a lateral line; and granules on each side. Tubercles of scapular setae *sc* on rear shield margin, 21 (19–21) apart, scapular setae *sc* 16 (15–16), divergently backward. **Coxae** with coarse distinct granules; prosternal apodeme 6 (5–6); setae *1b* 5 (5–6), tubercles *1b* 9 (7–9) apart; setae *1a* 17 (15–17), tubercles *1a* 8 (8–10) apart; setae *2a* 33 (30–35), tubercles *2a* 21 (20–22) apart. **Leg I** 26 (23–26), femur 8 (7–8), with fine granules, femoral setae *bv* 6 (5–6), genu 3 (3–4), genual setae *l*″ 15 (13–15), tibia 5 (4–5), tibial setae *l*′ 2*, tarsus 6 (5–6), tarsal setae *ft*′ 14 (12–15), setae *ft*″ 18 (15–18), setae *u*′ 3 (2–3), solenidion *ω* 7 (6–7) distally slightly knobbed, empodium simple, 6 (5–6), 4-rayed. **Leg II** 24 (23–25), femur 8 (7–8), with fine granules, femoral setae *bv* 5 (5–7), genu 3 (3), genual setae *l*″ 5 (5–6), tibia 4 (3–4), tarsus 5 (5–6), tarsal setae *ft*′ 4 (4–6), setae *ft*″ 15 (13–15), setae *u*′ 3 (2–3), solenidion *ω* 8 (7–8) distally slightly knobbed, empodium simple, 5 (5–6), 4-rayed. **Opisthosoma** with 68 (67–69) dorsal semiannuli, with elliptical microtubercles, and 65 (65–67) ventral semiannuli, with circular microtubercles on the rear annulus margin; coxigenital region with 4* semiannuli between coxae and genitalia, with circular microtubercles; spiny microtubercles on the rear margin of the last 10 (10–11) dorsal semiannuli; elongated and linear microtubercles on the last 6 ventral semiannuli. Setae *c2* 15 (15–16), on ventral semiannulus 11 (10–11), 39 (36–42) apart; setae *d* 35 (31–35), on ventral semiannulus 22 (22–23), 31 (27–33) apart; setae *e* 41 (36–45), on ventral semiannulus 38 (38–39), 17 (17–19) apart; setae *f* 12 (11–13), on 5th ventral semiannulus from rear, 14 (13–14) apart. Setae *h2* 48 (45–52), setae *h1* 4 (3–4). **Genital coverflap** 13 (12–14), 19 (18–21) wide, with some strong granulated lines at the genital coverflap base and 8–9 longitudinal ridges distally; setae *3a* 6 (5–6), 13 (11–13) apart. **Internal female genitalia**, transverse genital apodeme trapezoidal, with thickened anterior margin; longitudinal bridge relatively long; spermathecae bulbous; both spermathecae are equal in size; spermathecal tubes short, slightly swollen, directed posterolaterad.

**Male** (n = 3). Similar in shape and prodorsal shield arrangement to female. Body 175–188, 44–45 wide. **Gnathosoma** 15–16, projecting obliquely downwards, chelicerae 15*, setae *ep* 2*, setae *d* 2–3, unbranched, setae *v* 1–2. **Prodorsal shield** 26–28, 32–34 wide. Tubercles of the scapular setae *sc* ahead of rear shield margin 15–16 apart, setae *sc* 14–15. **Coxae** similar to that of the female; setae *1b* 5–6, tubercles *1b* 7–8 apart; setae *1a* 14–16, tubercles *1a* 9–10 apart; setae *2a* 26–28, tubercles *2a* 17–18 apart. **Leg I** 21–24, femur 7–8, femoral setae *bv* 5–6, genu 4*, genual setae *l*″ 14–16, tibia 3*, tibial setae *l*′ 2–3, tarsus 5*, tarsal setae *ft*′ 8–9, setae *ft*″ 15–18, setae *u*′ 3*, solenidion *ω* 6–7 slightly knobbed, empodium simple, 5–6, 4-rayed. **Leg II** 21–24, femur 7–8, femoral setae *bv* 4–5, genu 3–4, genual setae *l*″ 5*, tibia 4*, tarsus 5*, tarsal setae *ft*′ 4–6, setae *ft*″ 14–16, setae *u*′ 2*, solenidion *ω* 7* slightly knobbed, empodium simple, 5*, 4-rayed. **Opisthosoma** dorsally arched with 67–68 semiannuli, with elongate microtubercles on rear annular margins; 66–67 ventral semiannuli, with small circular microtubercles on rear annulus margin; 4* semiannuli between coxae and genital region. Setae *c2* 14–16 on ventral semiannulus 10*, 37–39 apart; setae *d* 30–32 on ventral semiannulus 20–21, 26–27 apart; setae *e* 33–35 on ventral semiannulus 36–38, 15–16 apart; setae *f* 10–11 on 6th ventral semiannulus from rear, 11–12 apart. Setae *h2* 40–46; setae *h1* 2*. Genitalia 12–13, 18–20 wide, setae *3a* 4*, 13–14 apart.

**Type material:** Holotype, female (slide number EAA2-5.1; marked Holotype), found on *Cryptocarya metcalfiana* Allen (Fam. Lauraceae), Mulun National Nature Reserve, Hechi City, Guangxi, China, 25°9′31″ N, 108°3′30″ E, elevation 306.7 m, 28 July 2021, coll. Meng-Chao Tan, An-Kang Lv. Paratypes, 14 females on 14 slides and three males on three slides (slide number EAA2-5.2~5.18; marked Paratypes), from *C. metcalfiana*, with the same details as holotype.

**Type of host plant:** *Cryptocarya metcalfiana* Allen (Fam. Lauraceae).

**Relation to the host plant:** causing the formation of erinea on the undersurface of the leaves, with slight bulges on the opposite side of the lamina. (Figure 3C,D)

**Etymology:** the species is named after the generic name of the type of host plant, i.e., *Cryptocarya* in the genitive case.

**Differential diagnosis:** *Aceria cryptocaryae* **sp. nov.** is most similar to *Aceria tribuli* (Keifer, 1974) collected from *Tribulus terrestris* L. (Zygophyllaceae) in Sudan and Egypt [24,25], in the prodorsal shield ornamentation pattern, sculpture of coxae, and coverflap. The new species is distinguishable from *A. tribuli* for the femur of legs (with fine granules in *A. cryptocaryae* **sp. nov.** versus smooth in *A. tribuli*), empodium (4-rayed in *A. cryptocaryae* **sp. nov.** versus 6-rayed in *A. tribuli*), the number of rings of the opisthosoma (67–69 dorsal semiannuli and 65–67 ventral semiannuli in *A. cryptocaryae* **sp. nov.** versus 70–80 dorsal semiannuli and 70–75 ventral semiannuli in *A. tribuli*), the length of scapular setae *sc* (15–16 μm in *A. cryptocaryae* **sp. nov.** versus 50–55 μm in *A. tribuli*), the opisthosomal setae *c2* (15–16 μm in *A. cryptocaryae* **sp. nov.** versus 32–45 μm in *A. tribuli*), setae *d* (31–35 μm in *A. cryptocaryae* **sp. nov.** *versus* 66–75 μm in *A. tribuli*).

**Figure 6:**
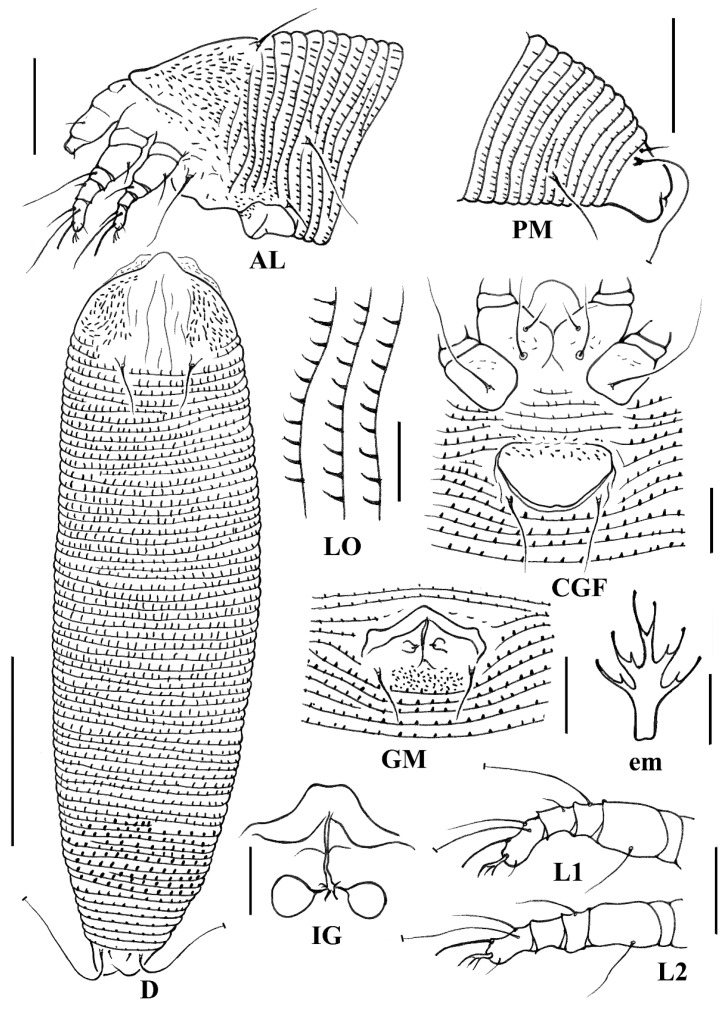
Line drawings of *Aceria buddlejae* **sp. nov.**: **AL**. Lateral view of anterior opisthosoma; **CGF**. Coxigenital region of female; **D**. Dorsal view; **em**. Empodium; **GM**. Male genitalia; **IG**. Internal female genitalia; **LO**. Lateral view of annuli; **L1**. Leg I; **L2**. Leg II; **PM**. Lateral view of the posterior opisthosoma. Scale bar: 50 μm for **D**; 25 μm for **AL** and **PM**; 10 μm for **CGF**, **GM**, **LO**, **L1**, **L2**, and **IG**; 2.5 μm for **em**.

**Figure 7:**
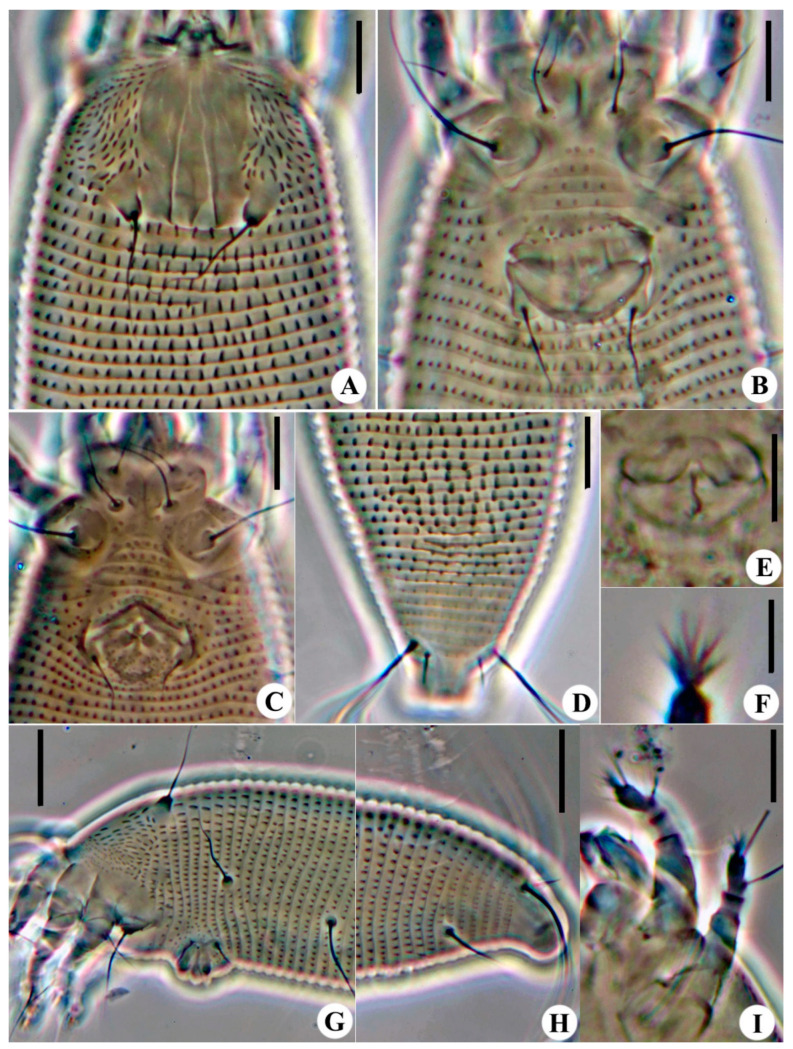
Images of *Aceria buddlejae* **sp. nov.**: (**A**) Prodorsal shield; (**B**) Female coxigenital area; (**C**) Male coxigenital area; (**D**) Postero-dorsal view of mite; (**E**) Internal genitalia; (**F**) Empodium; (**G**) Lateral view of anterior opisthosoma; (**H**) Postero-lateral view of mite; (**I**) Legs. Scale bar: 20 μm for G, H; 10 μm for (**A**–**E**), I; 5 μm for (**F**).

***Aceria buddlejae* sp. nov. (Figure 6 and Figure 7)**

Description: **Female** (n = 14). Body vermiform, 181 (170–195, including gnathosoma), 49 (45–49) wide, 48 (48–49) thick. **Gnathosoma** 19 (17–20), projecting obliquely downwards, setae *ep* 2 (2–3), setae *d* 5 (4–5), unbranched, setae *v* 1 (1–2), cheliceral stylets 20 (18–21). **Prodorsal shield** 29 (28–30), including frontal lobe, 39 (37–41) wide; short and rounded frontal lobe 4 (4–5) over gnathosomal base. Median lines are present on the posterior half of the shield; admedian lines are complete and sinuate; and submedian lines are present on the anterior half of the shield. Some short dashes and microtubercles are on the lateral sides of the shield. Tubercles of scapular setae *sc* on the rear shield margin, 17 (15–17) apart, scapular setae *sc* 15 (15–16), divergently backward. **Coxae** ornamented with some granules; prosternal apodeme 7 (6–7); setae *1b* 5 (5–6), tubercles *1b* 8 (7–9) apart; setae *1a* 13 (11–14), tubercles *1a* 9 (9–11) apart; setae *2a* 24 (23–26), tubercles *2a* 20 (19–22) apart. **Leg I** 25 (24–27), femur 7 (6–8), femoral setae *bv* 7 (5–8), genu 4*, genual setae *l*″ 18 (17–19), tibia 5 (4–5), tibial setae *l*′ 3 (3–4), tarsus 6 (6–7), tarsal setae *ft*′ 8 (6–9), setae *ft*″ 20 (17–20), setae *u*′ 3 (2–3), solenidion *ω* 7 (6–7) distally slightly knobbed, empodium simple, 5 (4–5), 3-rayed. **Leg II** 23 (23–26), femur 7 (7–8), femoral setae *bv* 8 (7–8), genu 3 (3–4), genual setae *l*″ 6 (5–7), tibia 4 (4–5), tarsus 6 (6–7), tarsal setae *ft*′ 4 (4–5), setae *ft*″ 19 (17–20), setae *u*′ 3 (2–3), solenidion *ω* 6 (6–7) distally slightly knobbed, empodium simple, 5 (4–5), 3-rayed. **Opisthosoma** with 65 (63–67) dorsal semiannuli, with elongate microtubercles on rear annular margins, and 67 (65–69) ventral semiannuli, with spiny microtubercles on rear annulus margin; coxigenital region with 4 (3–4) semiannuli between coxae and genitalia, with fine microtubercles; spiny microtubercles on rear margin of last 7 (7–8) dorsal semiannuli; elongated and linear microtubercles on last 8 ventral semiannuli. Setae *c2* 23 (21–23), on ventral semiannulus 12 (11–12), 41 (40–43) apart; setae *d* 45 (42–45), on ventral semiannulus 23 (22–23), 33 (31–34) apart; setae *e* 13 (11–13), on ventral semiannulus 40 (40–42), 19 (18–20) apart; setae *f* 18 (16–18), on 6th–7th ventral semiannulus from rear, 17 (17–18) apart. Setae *h2* 72 (60–77), setae *h1* 6 (5–6). **Genital coverflap** 12 (11–12), 18 (15–18) wide, with some strong granulated lines at the genital coverflap base; setae *3a* 13 (10–14), 14 (11–14) apart. **Internal female genitalia**, transverse genital apodeme trapezoidal, longitudinal bridge relatively long; oblique apodeme present; short spermathecal tubes, directed laterad; spermathecae oval-shaped, relatively small.

**Male** (n = 1). Similar in shape and prodorsal shield arrangement to female. Body 151*, 44* wide. **Gnathosoma** 19*, projecting obliquely downwards, chelicerae 21*, setae *ep* 2*, setae *d* 3*, unbranched, setae *v* 1*. **Prodorsal shield** 21*, 32* wide. Tubercles of the scapular setae *sc* ahead of the rear shield margin are 15* apart, setae *sc* 17*. **Coxae** are similar to those of the female; setae *1b* 6*, tubercles *1b* 8* apart; setae *1a* 13*, tubercles *1a* 7* apart; setae *2a* 27*, tubercles *2a* 18* apart. **Leg I** 26*, femur 7*, femoral setae *bv* 5*, genu 4*, genual setae *l*″ 17*, tibia 4*, tibial setae *l*′ 4*, tarsus 7*, tarsal setae *ft*′ 6*, setae *ft*″ 16*, setae *u*′ 3*, solenidion *ω* 6* slightly knobbed, empodium simple, 4*, 3-rayed. **Leg II** 25*, femur 7*, femoral setae *bv* 5*, genu 3*, genual setae *l*″ 4*, tibia 4*, tarsus 6*, tarsal setae *ft*′ 4*, setae *ft*″ 16*, setae *u*′ 3*, solenidion *ω* 7* slightly knobbed, empodium simple, 4*, 3-rayed. **Opisthosoma** dorsally arches with 61* semiannuli; 62* ventral semiannuli; 4* semiannuli between the coxae and genital region. Setae *c2* 19* on ventral semiannulus 10*, 35* apart; setae *d* 33* on ventral semiannulus 21*, 27* apart; setae *e* 13* on ventral semiannulus 35*, 15* apart; setae *f* 16* on ventral semiannulus 6th ventral semiannulus from rear, 16* apart. Setae *h2* 55*, setae *h1* 6*. Genitalia 11*, 14* wide, setae *3a* 8*, 11* apart.

**Type material:** Holotype, female (slide number EAA2-6.1; marked Holotype), found on *Buddleja lindleyana* Fort. (Fam. Scrophulariaceae), Chengkou County, Chongqing City, China, 32°16′29″ N, 108°46′75″ E, elevation 956.6 m, 27 August 2022, coll. Li-Mei Ren, An-Kang Lv. Paratypes, 12 females on 13 slides and one male on three slides (slide number EAA2-6.2~6.15; marked Paratypes), from *B. lindleyana*, with the same details as holotype.

**Type of host plant:** *Buddleja lindleyana* Fort. (Fam. Scrophulariaceae).

**Relation to the host plant:** vagrant on the leaves; no apparent damage was observed.

**Etymology:** the specific designation *buddlejae* is from the generic name of the host, *Buddleja*.

**Differential diagnosis:** *Aceria buddlejae* **sp. nov.** appears to be close to *Aceria noxia* Flechtmann and Tassi, 2020, that was found on *Amaranthus viridis* L. (Amaranthaceae), in the prodorsal shield ornamentation pattern, sculpture of coxae, seta *h1* present [26]. *Aceria buddlejae* **sp. nov.** can be differentiated from the above-mentioned species by the genitalia coverflap (with some strong granulated lines at the genital coverflap base in *A. buddlejae* **sp. nov.** *versus* coverflap basally with two transverse bands of coarse granules and distally with 14–16 longitudinal ridges in *A. noxia*), the frontal lobe (present in *A. buddlejae* **sp. nov.** versus absent in *A. noxia*), the number of empodium rays (3-rayed in *A. buddlejae* **sp. nov.** versus 5-rayed in *A. noxia*), the number of rings of the dorsal semiannuli (63–67 dorsal semiannuli in *A. buddlejae* **sp. nov.** versus 76–93 dorsal semiannuli in *A. noxia*), the length of the coxal seta III *2a* (23–26 μm in *A. buddlejae* **sp. nov.** versus 40–45 μm in *A. noxia*), the length of setae *c2* (21–23 μm in *A. buddlejae* **sp. nov.** versus 28–40 μm in *A. noxia*).

The new species is also similar to *Aceria hupehensis* Kuang and Hong, 1995, collected from *Castanea mollissima* Blume (Fagaceae) in China [27]. It shares the same prodorsal shield pattern, sculpture of coverflap, and number of empodium rays as *A. genistae*. However, the two species differ in: the frontal lobe (present in *A. tinctoriae* **sp. nov.** versus absent in *A. cumaniamajoris*), the number of rings of the opisthosoma (63–67 dorsal semiannuli and 65–69 ventral semiannuli in *A. buddlejae* **sp. nov.** versus 52–56 dorsal and ventral semiannuli in *A. hupehensis*), the length of setae *c2* (21–23 μm in *A. buddlejae* **sp. nov.** versus 6 μm in *A. hupehensis*), the length of setae *3a* (10–14 μm in *A. buddlejae* **sp. nov.** versus 5 μm in *A. hupehensis*).

## 4. Discussion

According to the data from the published references, there are 84 species (including 3 new species in this paper) of the genus *Aceria* that have been found in China, parasitizing 34 families and 77 species of plants [9]. Among them, 7 *Aceria* species of host plants belong to the family Salicaceae, and 5 *Aceria* species of host plants, respectively, belong to the families Poaceae and Solanaceae. In relation to the host plants of *Aceria*, 41 species cause galls on leaves, 8 species cause the formation of erinea on the undersurface of the leaves, and 29 species causes vagrants on the leaves. In terms of the geographical distribution and floral distribution of *Aceria*, there are 66 species in the Oriental realm and 24 species in the Palearctic realm. *Aceria kuko* (Kishida, 1927) and *Aceria lycopersici* (Massee, 1939) are distributed in most areas of China; *Aceria dispar* (Nalepa, 1891) and *Aceria tosichella* Keifer, 1969, are widely distributed in northern China; *Aceria litchii* (Keifer, 1943) and *Aceria hupehensis* Kuang and Hong, 1995, are widely distributed in southern China; others are only distributed in local areas. The discovery of three new *Aceria* species in China indicates that the species richness of the genus is still underestimated. Undoubtedly, it is necessary to further collect and investigate the taxa of *Aceria* in the future to understand their real diversity.

However, there are still some problems in the classification of the genus *Aceria*. Due to the limitations of early microscopic techniques and the low standards for the description of new species of *Aceria*, many *Aceria* species were not described in detail when they were published. The quality of illustrations was poor or no illustrations, the host plants were not identified, the damage description of *Aceria* to the host plants was simple, and the naming was irregular, which led to the emergence of a large number of homonyms in *Aceria* in recent years. For example, the *Aceria* species studied by Nalepa were stored in the Vienna Museum of Natural History in the United States. Due to long-term poor management, the alcohol-soaked specimens had dried up, the slide specimens could not be observed, the original manuscripts were lost, and many type specimens could not be verified [18,28]. Therefore, there are still some known *Aceria* species that need to be collected again for supplementary description and revision. In addition, most *Aceria* species are mainly distinguished by similar characteristics such as dorsal shield decoration, the number of dorsal and ventral rings, the genital coverflap decoration, the number of empodium, and so on. The distinguishing features of some closely related species are very subtle, and there may be certain differences on the same host in different regions [29,30,31]. Therefore, a combination of molecular and morphological methods is needed to determine the taxonomic status of species.

## 5. Conclusions

In this research, we described three new *Aceria* species: *Aceria bischofiae* **sp. nov.**, *Aceria cryptocaryae* **sp. nov.,** and *Aceria buddlejae* **sp. nov.** We also summarized the number of species, host plants, and geographical distribution of the genus *Aceria* in China. However, because *Aceria* species in northern China have not been systematically investigated yet, it is safe to assume that many other *Aceria* species may exist and will eventually be discovered.

## Data Availability

All data are available in this paper.

## References

[1] Lindquist E.E., Lindquist E.E., Sabelis M.W., Bruin J. (1996). External anatomy and notation of structures. Eriophyoid Mites—Their Biology, Natural Enemies and Control.

[2] Zhang Z.Q. (2017). Eriophyoidea and allies: Where do they belong?. Syst. Appl. Acarol..

[3] Skoracka A., Smith L., Oldfield G., Cristofaro M., Amrine J.W. (2010). Host-plant specificity and specialization in eriophyoid mites and their importance for the use of eriophyoid mites as biocontrol agents of weeds. Exp. Appl. Acarol..

[4] Yin Y., Yao L.F., Hu Y., Shao Z.K., Hong X.Y., Hebert P.D.N., Xue X.F. (2022). DNA barcoding uncovers cryptic diversity in minute herbivorous mites (Acari, Eriophyoidea). Mol. Ecol. Resour..

[5] Xue X.F., Yao L.F., Yin Y., Liu Q., Li N., Hoffmann A.A., Sun J.T., Yin Y., Hong X.Y. (2023). Macroevolutionary analyses point to a key role of hosts in diversification of the highly speciose eriophyoid mite superfamily. Mol. Phylogenet. Evol..

[6] Hong X.Y., Xue X.F., Song Z.W. (2010). Eriophyoidea of China: A review of progress, with a checklist. Zoosymposia.

[7] Liu L.X., Yao G., Yang J., Wang G.Q. (2022). One new genus, ten new species, and four new records of eriophyoid mites (Acari: Eriophyoidea) in Tibet, China. Syst. Appl. Acarol..

[8] Ruan H.Y., Hu L., Cui X.Y., Tan M.C. (2021). Three new species of eriophyoid mites (Acari: Eriophyoidea) associated with *Melicope pteleifolia* from Guangxi Zhuang Autonomous Region, China. Syst. Appl. Acarol..

[9] Xue X.F., Hong X.Y. (2023). A checklist of eriophyoid mites of China (Acariformes: Eriophyoidea). Syst. Appl. Acarol..

[10] Keifer H.H. (1944). Eriophyid studies XIV. Bull. Calif. Dep. Agric..

[11] Indra R., Bachheti R., Archana J. (2013). Chemical composition, mineral and nutritional value of wild *Bischofia javanica* seed. Int. Food Res. J..

[12] Boczek J., Chandrapatya A. (2000). Studies on eriophyoid mites (Acari: Eriophyoidea). XLI. Bull. Pol. Acad. Sci.-Tech..

[13] Wei S.G., Wang G.Q., Li D.W., Ou S.S. (2009). Eriophyoid Mites of Guangxi, China (Acari: Eriophyoidea).

[14] Liu S.S., Feng S.Y., Huang Y.Y., An W.L., Yang Z.R., Xie C.Z., Zheng X.S. (2021). Characterization of the Complete Chloroplast Genome of *Buddleja lindleyana*. J. AOAC Inter..

[15] Meyer M.K.P.S., Ueckermann E.A. (1990). African Eriophyidae: Genus *Aculops* Keifer 1966 (Acari: Eriophyidae). Phytophylactica.

[16] Nalepa A. (1923). Eriophyiden aus Java (4. Beitrag). Treubia.

[17] Amrine J.W., Manson D.C.M., Lindquist E.E., Sabelis M.W., Bruin J. (1996). Preparation, mounting and descriptive study of Eriophyoid mites. Eriophyoid Mites. Their Biology, Natural Enemies and Control.

[18] de Lillo E., Craemer C., Amrine J.W., Nuzzaci G. (2010). Recommended procedures and techniques for morphological studies of Eriophyoidea (Acari: Prostigmata). Exp. Appl. Acarol..

[19] Amrine J.W., Stasny T.A.H., Flechtmann C.H.W. (2003). Revised Key to the World Genera of Eriophyoidea (Acari: Prostigmata).

[20] Chetverikov P.E. (2014). Comparative confocal microscopy of internal genitalia of phytoptine mites (Eriophyoidea, Phytoptidae): New generic diagnoses reflecting host-plant associations. Exp. Appl. Acarol..

[21] Nalepa A. (1892). Neue Arten der Gattung *Phytoptus* Duj. und *Cecidophyes* Nal. Denkschriften kaiser Akademie der Wissenschaften: Mathematisch-Naturwissenschaftliche Classe.

[22] Mehri-Heyran H., Lotfollahi P., de Lillo E., Azimi S. (2020). Redescription of *Aceria varia* and *Tegoprionus dentatus* (Trombidiformes: Eriophyoidea: Eriophyidae) from Iran. Pers. J. Acarol..

[23] Kuang H.Y., Yang S.P., Lu Q.B. (1994). Four new species of the genus *Aceria* from Guangxi, China (Acari: Eriophyoidae). Acta Zootaxonomica Sin..

[24] Keifer H.H. (1974). Eriophyid Studies C-9.

[25] Elhalawany A.S., Mohamed A.A., Ueckermann E.A. (2022). Two new species and complementary descriptions of four new records of family Eriophyidae (Acari: Trombidiformes) in Egypt. Syst. Appl. Acarol..

[26] Flechtmann C.H.W., Tassi A.D. (2020). An *Aceria* species (Prostigmata, Eriophyidae) from *Amaranthus* in Brazil. Pers. J. Acarol..

[27] Kuang H.Y. (1995). Economic Insect Fauna of China. Fasc. 44. (Acari: Eriophyoidea) (I).

[28] Chetverikov P.E., Hörweg C., Kozlov M.I., Amrine J.W. (2016). Reconditioning of the Nalepa collection of eriophyoid mites (Acariformes, Eriophyoidea). Syst. Appl. Acarol..

[29] Lewandowski M., Skoracka A., Szydło W., Kozak M., Druciarek T., Griffiths D.A. (2014). Genetic and morphological diversity of *Trisetacus* species (Eriophyoidea: Phytoptidae) associated with coniferous trees in Poland: Phylogeny, barcoding, host and habitat specialization. Exp. Appl. Acarol..

[30] Laska A., Majer A., Szydło W., Karpicka-Ignatowska K., Hornyák M., Labrzycka A., Skoracka A. (2018). Cryptic diversity within grass-associated *Abacarus* species complex (Acariformes: Eriophyidae), with the description of a new species, *Abacarus plumiger* n. sp.. Exp. Appl. Acarol..

[31] Li H.S., Xue X.F., Hong X.Y. (2014). Cryptic diversity in host-associated populations of *Tetra pinnatifidae* (Acari: Eriophyoidea): What do morphometric, mitochondrial and nuclear data reveal and conceal?. Bull. Entomol. Res..

